# Sporadic late-onset nemaline myopathy: clinico-pathological characteristics and review of 76 cases

**DOI:** 10.1186/s13023-017-0640-2

**Published:** 2017-05-11

**Authors:** Lukas J. Schnitzler, Tobias Schreckenbach, Aleksandra Nadaj-Pakleza, Werner Stenzel, Elisabeth J. Rushing, Philip Van Damme, Andreas Ferbert, Susanne Petri, Christian Hartmann, Antje Bornemann, Andreas Meisel, Jens A. Petersen, Thomas Tousseyn, Dietmar R. Thal, Jens Reimann, Peter De Jonghe, Jean-Jacques Martin, Peter Y. Van den Bergh, Jörg B. Schulz, Joachim Weis, Kristl G. Claeys

**Affiliations:** 1 Department of Neurology, University Hospital RWTH Aachen, Aachen, Germany; 2 Institute of Neuropathology, University Hospital RWTH Aachen, Aachen, Germany; 3 Department of Neurology, Medizinisches Zentrum StädteRegion Aachen, Würselen, Germany; 4 Reference Centre for Neuromuscular Diseases, Department of Neurology, University Hospital Angers, Angers, France; 5 Department of Neuropathology, Charité - Universitätsmedizin Berlin, Berlin, Germany; 6 Institute of Neuropathology, University Hospital Zürich, Zürich, Switzerland; 7 Department of Neurology, University Hospitals Leuven, Leuven, Belgium; 8 Department of Neurosciences, Experimental Neurology, University of Leuven (KU Leuven), Leuven, Belgium; 9 VIB, Center for Brain and Disease Research, Leuven, Belgium; 10 Department of Neurology, Klinikum Kassel, Kassel, Germany; 11 Department of Neurology, Hannover Medical School, Hannover, Germany; 12 Department of Neuropathology, Hannover Medical School, Hannover, Germany; 13 Institute of Neuropathology, University Hospital Tübingen, Tübingen, Germany; 14 Department of Neurology, University Hospital Charité Berlin, Berlin, Germany; 15 Department of Neurology, University Hospital Zurich and University of Zurich, Zurich, Switzerland; 16 Department of Pathology, University Hospitals Leuven, Leuven, Belgium; 17 Department of Neurosciences, Experimental Neurology, Laboratory for Neuropathology, University of Leuven (KU Leuven), Leuven, Belgium; 18 Department of Neurology, University Hospital Bonn, Bonn, Germany; 19 Department of Neurology, University Hospital Antwerpen, Antwerpen, Belgium; 20 Institute of Molecular Genetics (VIB), University of Antwerpen, Antwerpen, Belgium; 21 Born-Bunge Institute, University of Antwerpen, Antwerpen, Belgium; 22 Department of Neurology, University Hospital Saint-Luc, Brussels, Belgium; 23 JARA-BRAIN Institute Molecular Neuroscience and Neuroimaging, Forschungszentrum Jülich GmbH and RWTH Aachen University, Aachen, Germany; 24 Laboratory for Muscle Diseases and Neuropathies, Department of Neurosciences, Experimental Neurology, KU Leuven - University of Leuven, Leuven, Belgium; 25 Campus Gasthuisberg, Department of Neurology, University Hospitals Leuven and KU Leuven (University of Leuven), Herestraat 49, B-3000 Leuven, Belgium

**Keywords:** SLONM, Muscle biopsy, HIV-associated nemaline myopathy, HIV-NM, Monoclonal gammopathy, MGUS, NGS

## Abstract

**Background:**

Sporadic late-onset nemaline myopathy (SLONM) is a rare, late-onset muscle disorder, characterized by the presence of nemaline rods in muscle fibers. Phenotypic characterization in a large cohort and a comprehensive overview of SLONM are lacking.

**Methods:**

We studied the clinico-pathological features, treatment and outcome in a large cohort of 76 patients with SLONM, comprising 10 new patients and 66 cases derived from a literature meta-analysis (PubMed, 1966–2016), and compared these with 15 reported HIV-associated nemaline myopathy (HIV-NM) cases. In 6 SLONM patients, we performed a targeted next-generation sequencing (NGS) panel comprising 283 myopathy genes.

**Results:**

SLONM patients had a mean age at onset of 52 years. The predominant phenotype consisted of weakness and atrophy of proximal upper limbs in 84%, of proximal lower limbs in 80% and both in 67%. Other common symptoms included axial weakness in 68%, as well as dyspnea in 55% and dysphagia in 47% of the patients. In 53% a monoclonal gammopathy of unknown significance (MGUS) was detected in serum. The mean percentage of muscle fibers containing rods was 28% (range 1–63%). In 2 cases ultrastructural analysis was necessary to detect the rods. The most successful treatment in SLONM patients (all with MGUS) was autologous peripheral blood stem cell therapy. A targeted NGS gene panel in 6 SLONM patients (without MGUS) did not reveal causative pathogenic variants.

In a comparison of SLONM patients with and without MGUS, the former comprised significantly more males, had more rapid disease progression, and more vacuolar changes in muscle fibers. Interestingly, the muscle biopsy of 2 SLONM patients with MGUS revealed intranuclear rods, whereas this feature was not seen in any of the biopsies from patients without paraproteinemia.

Compared to the overall SLONM cohort, significantly more HIV-NM patients were male, with a lower age at onset (mean 34 years). In addition, immunosuppression was more frequently applied with more favorable outcome, and muscle biopsies revealed a significantly higher degree of inflammation and necrosis in this cohort. Similar to SLONM, MGUS was present in half of the HIV-NM patients.

**Conclusions:**

SLONM presents a challenging, but important differential diagnosis to other neuromuscular diseases of adult onset. Investigations for MGUS and HIV should be performed, as they require distinct but often effective therapeutic approaches. Even though SLONM and HIV-NM show some differences, there exists a large clinico-pathological overlap between the 2 entities.

**Electronic supplementary material:**

The online version of this article (doi:10.1186/s13023-017-0640-2) contains supplementary material, which is available to authorized users.

## Background

Sporadic late-onset nemaline myopathy (SLONM) is a rare, acquired, late-onset muscle disorder with subacute progression, characterized by proximal muscle weakness and atrophy, and the presence of nemaline rods in myofibers [1, 2]. Distal muscle weakness [3], dropped head [4, 5], respiratory failure [6, 7], or dysphagia [8, 9] have also been reported. SLONM can be associated with a monoclonal gammopathy of unknown significance (MGUS) that leads to an unfavorable outcome if untreated [10–13].

HIV-associated nemaline myopathy (HIV-NM) is another acquired, late-onset nemaline myopathy, distinguished by the presence of nemaline rods in muscle fibers in the setting of an HIV-infection [14–16]. An autoimmune mechanism for HIV-NM has been discussed [17, 18], however, it remains unclear whether HIV-NM and SLONM represent the same or distinct disease entities.

Histopathologically, nemaline rods can be identified on modified Gomori’s trichrome staining (mGT) as subsarcolemmal or intermyofibrillar dark red clusters [19]. Using electron microscopy, the rods appear as electron dense bodies, sometimes showing continuity with the Z-discs [20]. Rarely, nemaline bodies can occur inside myonuclei [4, 12, 21, 22]. Rods also represent the histopathological hallmark of the inherited congenital nemaline myopathies, which are currently known to be caused by 11 different genes [23].

Several case reports and small series of patients with SLONM have been published so far, but a systematic overview of SLONM is lacking. Here, we studied the clinico-pathological features as well as treatment and outcome in a large cohort of 76 patients with SLONM, comprising 10 new patients and 66 cases derived from a literature meta-analysis. In addition, we screened 283 myopathy genes using a targeted next-generation sequencing (NGS) panel in 6 SLONM patients and compared the phenotypic characteristics of the SLONM cohort (*n* = 76) with 16 HIV-NM cases reported in the literature.

## Methods

### SLONM patient selection

Patients with histologically confirmed SLONM, who either presented at the neuromuscular outpatient clinic (Department of Neurology) or had a muscle biopsy sent for (re-) evaluation to the Institute of Neuropathology at the University Hospital RWTH Aachen (Aachen, Germany), were included in our study. Patients with nemaline myopathy and a history or symptoms suggesting a congenital myopathy, such as positive family history for myopathy, the presence of delayed motor milestones, high-arched palate, scoliosis or muscle hypotonia were excluded. Patients with HIV infection were also excluded.

### Clinical and paraclinical examinations

We performed a detailed neurological examination including muscle strength evaluation according to the criteria of the Medical Research Council (MRC) scale. Blood analyses were obtained including creatine kinase (CK) (normal in men <190 U/l, women <170 U/l), immune fixation and thyroid function as well as electromyography (EMG), nerve conduction studies (NCS), vital capacity measurement using spirometry, and echocardiography in all patients (Table 1, P1-10). The same examinations were performed in two patients (Table 1, P11-P12) who had been previously reported [9, 24], but who were re-evaluated in the current study and included for genetic analysis (see below).

**Table 1:** Clinical, paraclinical and muscle biopsy features in SLONM, and comparison with HIV-NM

	SLONM															HIV-NM
	All SLONM cases (76)	With gammopathy (27/76)	Without gammopathy (24/76)	P 1	P 2	P 3	P 4	P 5	P 6	P 7	P 8	P 9	P 10	P 11	P 12	All HIV-NM cases (15)
History:																
Gender	35 m/33f ^a^	**18 m/9f**	**7 m/17f**	m	f	f	f	f	f	m	f	m	f	f	f	13 m/2f ^a^
Age at onset (mean/range) (y)	52/25–78 ^a^	49/25–78	53/27–78	52	67	61	49	54	32	50	47	51	77	67	54	34/21–49 ^a^
Age at examination (mean/range) (y)	56/27–79	51/26–75	58/34–79	55	72	67	49	79	34	51	48	52	79	79	60	34/21–49
Duration until diagnosis (mean/range) (y)	4/0–32	2/0–7	4/0–32	3	5	5	4	25	3	1	3	1	2	2	6	1/0–4
Stable/slow progression	42/50 84%	9/18 50%	17/17 100%	**+**	**+**	**+**	**+**	**+**	**+**	**+**	**-**	**-**	**+**	**+**	**+**	6/10 60%
Rapid progression	8/50 16%	**9/18 50%**	**0/16 0%**	**-**	**-**	**-**	**-**	**-**	**-**	**-**	**+**	**+**	**-**	**-**	**-**	3/10 40%
Preceding autoimmune diseases	8/76 11%	1/27 4%	5/24 21%	**-**	**-**	**+**	**-**	**-**	**-**	**-**	**-**	**-**	**+**	**-**	**-**	0/15 0%
Clinical features (in %):																
Weakness:																
Neck extensor	31/56 (55)	15/25 (60)	9/20 (45)	**-**	**-**	**-**	**+**	**-**	**-**	**+**	**+**	**-**	**-**	**+**	**+**	3/3 (100)
Neck flexor	22/36 (61)	6/11 (55)	8/13 (62)	**-**	**-**	**-**	**-**	**+**	**-**	**+**	**+**	**-**	**-**	**+**	**+**	1/1 (100)
Abdominal/back	17/25 (68)	8/10 (80)	6/9 (67)	**-**	**-**	**+**	**-**	**-**	**-**	**+**	**+**	**-**	**+**	**+**	-	2/2 (100)
Upper limbs	43/52 (83)	**19/19 (100)**	**11/17 (65)**	**+**	**-**	**+**	**+**	**-**	**-**	**+**	**+**	**+**	**-**	**+**	**-**	11/11 (100)
Lower limbs	40/49 (82)	**17/18 (94)**	**9/15 (60)**	**+**	**+**	**+**	**-**	**+**	**-**	**-**	**+**	**+**	**-**	**+**	**-**	8/8 (100)
Proximal	59/64 (92)	27/27 (100)	18/23 (78)	**+**	**-**	**+**	**+**	**+**	**-**	**+**	**+**	**+**	**-**	**+**	**-**	15/15 (100)
Predominantly proximal	43/59 (73)	25/27 (93)	14/18 (78)	**+**	**-**	**+**	**+**	**+**	**-**	**+**	**+**	**+**	**-**	**+**	**-**	14/15 (93)
Distal	31/59 (53)	11/22 (50)	10/23 (44)	**+**	**+**	**-**	**-**	**-**	**-**	**-**	**+**	**-**	**-**	**+**	**-**	4/15 (27)
Predominantly distal	4/31 (13)	0/11 (0)	2/10 (20)	**-**	**+**	**-**	**-**	**-**	**-**	**-**	**-**	**-**	**-**	**-**	**-**	0/4 (0)
Facial weakness	17/48 (35)	7/19 (37)	2/17 (12)	**-**	**-**	**-**	**-**	**-**	**-**	**-**	**-**	**-**	**-**	**-**	**+**	0/4 (0)
Ophthalmoparesis	1/21 (5)	0/7 (0)	1/8 (13)	**-**	**-**	**-**	**-**	**-**	**-**	**-**	**-**	**-**	**-**	**-**	**+**	0/4 (0)
Ptosis	2/16 (13)	1/6 (17)	1/8 (13)	**-**	**-**	**-**	**-**	**-**	**-**	**-**	**-**	**-**	**-**	**-**	**+**	0/4 (0)
Dysphagia	21/45 (47)	12/22 (55)	5/16 (31)	**+**	**-**	**-**	**+**	**-**	**+**	**-**	**-**	**-**	**-**	**-**	**+**	1/5 (20)
Dysarthria	6/17 (35)	4/8 (50)	1/7 (14)	**+**	**-**	**-**	**-**	**-**	**-**	**-**	**-**	**-**	**-**	**-**	**+**	0/0 (0)
Dyspnea	17/31 (55)	9/13 (69)	3/7 (43)	**+**	**-**	**+**	**+**	**-**	**+**	**-**	**-**	**-**	**-**	**-**	-	0/1 (0)
Muscle atrophy	47/53 (89)	23/24 (96)	15/18 (83)	**+**	**-**	**-**	**+**	**-**	**-**	**+**	**-**	**+**	**+**	**+**	**+**	6/6 (100)
Myalgia	20/47 (43) ^a^	5/17 (29)	5/17 (29)	**-**	**-**	**+**	**+**	**+**	**+**	**+**	**-**	**-**	**-**	**+**	-	4/4 (100) ^a^
Cramps	5/14 (36)	1/4 (25)	1/7 (14)	**-**	**-**	**-**	**-**	**-**	**-**	**-**	**-**	**-**	**-**	**+**	-	0/0 (0)
Fasciculations	2/13 (15)	1/6 (17)	0/7 (0)	**-**	**-**	**-**	**-**	**-**	**-**	**-**	**-**	**-**	**-**	**-**	-	0/0 (0)
Deep tendon reflexes:																
normal	15/38 (11)	6/12 (50)	5/13 (39)	**-**	**-**	**-**	**+**	**+**	**+**	**-**	**+**	**-**	**+**	**-**	-	5/8 (63)
increased	4/38 (39)	1/12 (8)	2/13 (15)	**-**	**-**	**-**	**-**	**-**	**-**	**-**	**-**	**+**	**-**	**-**	+	0/8 (0)
decreased or absent	19/38 (50)	5/12 (42)	6/13 (46)	**+**	**+**	**+**	**-**	**-**	**-**	**+**	**-**	**-**	**-**	**+**	-	3/8 (38)
Paraclinical examination (in %):																
IgG gammopathy present	27/51 (53)	27/27 (100)	0/24 (0)	**+**	**-**	**-**	**+**	NA	**-**	**-**	**+**	**+**	**-**	**-**	**-**	3/6 (50)
kappa	14/27 (52)	14/27 (52)	0/24 (0)	**+**	**-**	**-**	**-**	NA	**-**	**-**	**-**	**+**	**-**	**-**	**-**	1/3 (33)
lambda	11/27 (41)	11/27 (41)	0/24 (0)	**-**	**-**	**-**	**+**	NA	**-**	**-**	**+**	**-**	**-**	**-**	**-**	2/3 (67)
kappa + lambda	1/27 (4)	1/27 (4)	0/24 (0)	**-**	**-**	**-**	**-**	NA	**-**	**-**	**-**	**-**	**-**	**-**	**-**	0/3 (0)
multiple myeloma	1/27 (4)	1/27 (4)	0/20 (0)	**-**	**-**	**-**	**-**	NA	**-**	**-**	**-**	**-**	**-**	**-**	**-**	0/0 (0)
CK with mean/range [number of patients examined]	2.6/1–55 N [60/76]	1.5 N/1–4 N [24/27]	1.9 N/1–17 N [23/24]	N	N	2 N	3 N	NA	2 N	17 N	2 N	N	N	2 N	N	1.8 N/1–5 N [13/15]
Respiratory insufficiency	11/20 (55)	6/9 (67)	3/8 (38)	+	NA	**-**	**+**	**-**	**+**	**-**	**-**	**-**	**-**	**-**	-	0/0 (0)
Cardiomyopathy	3/19 (16)	1/5 (20)	2/7 (29)	**-**	NA	**-**	dil.	-	dil.	-	**-**	**-**	**-**	**-**	-	0/0 (0)
Electromyogram																
normal	2/63 (3)	0/27 (0)	2/21 (10)	**-**	**-**	**-**	**-**	**-**	**-**	**-**	**-**	**-**	**+**	**-**	-	0/12 (0)
myopathic	53/63 (84)	24/27 (89)	16/21 (76)	**-**	**+**	**-**	**+**	**+**	**+**	**+**	**+**	**+**	**-**	**+**	+	12/12 (100)
mixed	6/63 (10)	3/27 (11)	2/21 (10)	**+**	**-**	**-**	**-**	**-**	**-**	**-**	**-**	**-**	**-**	**-**	-	0/12 (0)
neurogenic	2/63 (3)	0/27 (0)	1/21 (5)	**-**	**-**	**+**	**-**	**-**	**-**	**-**	**-**	**-**	**-**	**-**	-	0/12 (0)
Genetic panel analysis performed				**-**	**+**	**-**	**-**	**-**	**+**	**+**	**-**	**-**	**+**	**+**	+	
Muscle biopsy:				VL	TA	VL	D	GM	D	QF	VL	QF	VL	BF	SC	
% of rod containing cells with mean/range [number of patients with available data]	28/1–63 [36/76]	30/3–63 [12/27]	22/1–50 [14/24]	3	44	3	43	NA	24	NA	16	28	5	14	35	45/35–50 [3/15]
Intranuclear rods	4/24 (17)	2/9 (22)	0/9 (0)	**-**	**-**	**-**	**-**	NA	-	-	-	-	**-**	-	**-**	0/0 (0)
Muscle fiber atrophy	45/45 (100)	15/15 (100)	13/13 (100)	**+**	**+**	**+**	**+**	**+**	**+**	+	+	+	**+**	**+**	**+**	14/14 (100)
Internalized nuclei	27/37 (73)	10/13 (77)	5/11 (46)	**+**	+	**+**	**+**	**+**	-	-	+	-	**-**	-	-	7/7 (100)
Myofibrillar disintegration	21/24 (88)	8/8 (100)	6/9 (67)	**+**	**+**	**-**	**+**	NA	+	**+**	**+**	**+**	**-**	+	-	2/2 (100)
Lobulated/trabeculated fibers	17/33 (52)	7/14 (50)	9/19 (50)	**-**	**+**	**-**	**+**	**+**	**-**	-	-	-	**-**	-	**+**	1/1 (100)
Endomysial fibrosis	17/26 (65)	6/8 (75)	4/9 (44)	**+**	+	**-**	**+**	NA	+	-	+	-	**-**	-	+	1/1 (100)
Inflammatory infiltrates	9/53 (17)	3/17 (18)	2/22 (9)	**-**	-	**-**	**-**	**-**	-	**-**	**-**	**-**	**-**	-	-	6/14 (43)
Necrotic fibers	7/28 (25) ^a^	3/10 (30)	2/12 (17)	**+**	-	**-**	-	NA	+	+	-	-	**-**	-	-	4/5 (80) ^a^
Core-like areas	11/16 (69)	5/6 (83)	3/7 (43)	**+**	+	**+**	-	NA	**-**	+	+	+	**-**	-	-	0/0 (0)
Vacuolar changes	11/20 (55)	**5/7 (71)**	**1/8 (13)**	+	**-**	**-**	+	**+**	-	+	-	-	**-**	+/−	-	5/5 (100)
Mitochondrial changes	11/18 (61)	2/5 (40)	3/7 (17)	-	-	unsp.	-	**+**	-	**-**	**+**	**-**	unsp.	unsp.	-	1/2 (50)
Therapy (and favorable outcome):																
Immunosuppressive agents	36/58 (12/36) ^a^	19/24 (6/19)	9/17 (2/9)	+ (+)	**-**	**-**	+ (−)	**-**	**-**	+ (+)	+ (+)	+ (NA)	**-**	**-**	-	8/10 (8/8) ^a^
Intravenous Immunoglobulins	11/58 (5/11)	7/24 (3/7)	4/17 (2/4)	**-**	**-**	**-**	**-**	**-**	**-**	+ (+)	-	-	**-**	**-**	-	2/10 (2/2)
Stem cell therapy	7/58 (6/7)	7/24 (6/7)	0/17 (0/0)	**-**	**-**	**-**	**-**	**-**	**-**	**-**	**+ (+)**	+ (NA)	**-**	**-**	-	0/10
Plasmapheresis	3/58 (1/3)	2/24 (0/2)	0/17 (0/0)	**-**	**-**	**-**	+ (−)	**-**	**-**	**-**	**-**	**-**	**-**	**-**	-	1/10 (1/1)
Noninvasive ventilation	3/58	0/24	2/17	**+**	**-**	**-**	**-**	**-**	**+**	**-**	**-**	**-**	**-**	**-**	-	0/10

In bold, statistically significant differences between SLONM with and without monoclonal gammopathy are indicated

*HIV-NM* HIV-associated nemaline myopathy, *m* male, *f* female, *y* years, + symptom/feature present, *NA* not available, − symptom/feature not present, *IgG* immunoglobulin G, *CK* serum creatine kinase, *N* normal value (men <190 U/l, women <170 U/l), *dil*. dilated cardiomyopathy, *VL* musculus vastus lateralis, *TA* musculus tibialis anterior, *D* musculus deltoideus, *GM* musculus gluteus medius, *QF* musculus quadriceps femoris, *BF* musculus biceps femoris, *SC* musculus sternocleidomastoideus, u*nsp.* unspecific mitochondrial changes

^a^ indicates statistically significant differences between all SLONM cases and HIV-NM

### Histopathological examination

Open muscle biopsies had been performed for diagnostic purposes prior to the study after obtaining the patients’ written informed consent (Table 1, P1-10). Biopsies were processed for light microscopy (LM) and electron microscopy (EM) following standardized procedures [19]. Electron microscopic studies were performed in 49 published cases and all of our new patients but patient 4, in whom only LM was available. All available LM sections and EM images were analyzed. Biopsies from two additional patients (Table 1, P11-P12) who had been previously reported [9, 24] were re-evaluated in the current study.

### Statistical analysis

Statistical analysis was performed using Excel software (Version 16.0.7030.1021) with the Add-In Real Statistics Resource Pack (Release 4.3). We tested for normal distributions using the D’Agostino Pearson Omnibus and the Shapiro-Wilk normality tests and compared normally distributed independent samples using Welch’s *t*-test, and non-normally distributed independent samples using the Mann-Whitney *U* test. For categorical variables, the two-tailed Fisher’s exact test was applied. *P*-values less than 0.05 were considered to be statistically significant.

### Targeted next-generation sequencing (NGS) gene panel

In 6 SLONM patients without MGUS, targeted NGS was performed using a focused panel of 283 genes (CeGat, Tübingen, Germany). After obtaining written informed consent from each patient, peripheral blood samples were taken and DNA was extracted following standard procedures. The gene panel contained 73 genes that have so far been associated with congenital or distal myopathies, including genes known to cause congenital nemaline myopathy. Of these 73 genes, all variants were further considered. In addition, 210 genes related to diverse myopathies were included, in which only variants that were either marked as “known pathogenic” in the Human Genome Mutation Database (HGMD®), produced a STOP codon, resulted in a frameshift or were located in an essential splice site, were taken into further consideration. The applied prediction programs were Mutation Taster (MT), PolyPhen-2 (PP-2), PROVEAN, SIFT, Splice-Prediction Program NG2: NetGene2 Server, and Splice-Site Prediction by Neural Network.

### Meta-analysis of the literature

We performed a systematic literature search in PubMed for SLONM cases from 1966 to 2016. All articles written in English, German and French were considered. The same inclusion and exclusion criteria that were used in our study were applied, which resulted in 46 publications comprising 66 SLONM patients in total (Table 1). In order to compare the features of SLONM patients with HIV-associated nemaline myopathy (HIV-NM), we performed an additional literature search for patients with HIV-NM. This resulted in 10 publications comprising 16 cases of HIV-NM (Table 1).

## Results

### Clinical and paraclinical features of patients with SLONM (Table 1)

Our SLONM study cohort comprised 10 new patients and 66 cases culled from the literature (Table 1). Mean age at symptom onset was 52 years, ranging from 25 to 78 years. In half of the patients, first symptoms occurred between 45 and 60 years of age. Sixty percent were diagnosed within the first 2 years after symptom onset, while the longest diagnostic delay was 32 years (Table 1).

At disease onset, 80% of the patients presented with muscle weakness of the proximal lower and/or upper limbs. Neck extensor weakness (dropped head) or dyspnea were the only presenting symptoms in four cases each (8%). In two patients, isolated dysphagia marked the beginning of the disease (4%). During the disease course, the predominant clinical phenotype consisted of weakness and atrophy affecting the proximal upper limbs in 84%, proximal lower limbs in 80%, and both proximal upper and lower limbs in 67% as well as axial weakness (68%). Dyspnea developed in 55%, dysphagia in 47% of the patients. Muscle weakness progressed slowly in most SLONM patients. In one third, there was evidence of facial weakness (35%) or dysarthria (35%). Myalgia occurred in 43%, cramps in 36%, and fasciculations in 15%. Other symptoms presented more infrequently: predominantly distal weakness was seen in four patients (13%), ptosis in two patients (13%), and ophthalmoparesis was noted in one patient (5%) (Table 1).

In 53% of patients with SLONM, an MGUS (IgG) was detected in serum (Table 1). Urine investigations (performed in 5 patients) did not reveal the presence of monoclonal immunoglobulins, except for one case (however, without underlying hematological malignancy) [25]. Fifty-two percent of the MGUS patients had kappa light chains, 41% lambda, and one patient presented with both kappa and lambda light chains in the serum [26]. An underlying multiple myeloma was found in only one case [27]. Serum CK was mostly normal (72%) or only slightly increased (<3 N; 22%). Three cases presented with hypothyroidism, which could be explained by amiodarone intake in one patient (P4). In the other two, anti-thyroid peroxidase antibodies and anti-thyroglobulin antibodies were negative in serum. TSH was increased in one [28] and decreased in another [29] patient. Electromyography (EMG) mainly showed myopathic changes (84%). Ten percent of patients showed a mixed pattern on EMG, two patients presented a purely neurogenic (3%) ((P3) and [30]) and two more a normal pattern (3%) ((P9) and [31]). Respiratory insufficiency was detected by spirometry in 55%. Dilated cardiomyopathy was present in 11% of patients.

### Muscle biopsy findings in patients with SLONM (Table 1)

Nemaline bodies were visible by LM in all except 2 biopsies (P2, P7), in which EM was necessary to detect the rods, which were situated in the sarcoplasm and/or underneath the sarcolemma. Interestingly, intranuclear rods were present in 17% of the cases, but always combined with intrasarcoplasmic rods. The mean percentage of muscle fibers containing rods was 28%, varying from 1 to 63% (Table 1). Muscle fiber atrophy was present in all biopsies. Further frequent histopathological findings included myofibrillar disintegration (88%), core-like areas (69%), lobulated fibers (52%) and vacuolar changes (55%). Fiber necrosis was present in 25% and inflammatory infiltrates were found in 17% of the biopsies. The infiltrates were restricted to perivascular areas in 2 cases, whereas additional more widespread endomysial infiltrates were observed in the others. Mitochondrial changes (ragged-red fibers, COX-negative fibers and/or paracrystalline inclusions on EM) occurred frequently (64%). However, these could be explained at least in part as an unspecific age-related phenomenon.

### Treatment in patients with SLONM (Tables 1 and 2)

**Table 2:** Treatment and outcome in patients with SLONM (novel patients P1-10 and literature data)

Ref.	Treatment	Outcome
P 1 ^a^	steroids + NIV	R: improvement of limb and respiratory strength
P 2	NA	NA
P 3	analgetics + physiotherapy	NA
P 4 ^a^	steroids + plasmapheresis	NR, cardiac decompensation
	cyclophosphamide + rituximab	R: stabilisation of weakness
P 5	none	NA
P 6	NIV	NA
P 7	IVIg + steroids	R: increased muscle strength, CK remained increased
P 8 ^a^	auto-PBSCT + melphalan + dexamethasone + lenalidomide	R: increased muscle strength
P 9 ^a^	auto-PBSCT + steroids	NA
P 10	none	NA
[24] (P 11)	none	NA
[9] (P 12)	none	NA
[54] ^a^	auto-PBSCT + melphalan	R: paraprotein in serum + rods disappeared, increased muscle strength
[12] ^a^	steroids	NR: died within 1y
[12] ^a^	steroids	NR: died within 1y
[12]	steroids	NR
[12]	steroids	NR: stable
[12] ^a^	steroids	NR: died within 1y
[12]	steroids	NR: stable 14y after onset
[12]	steroids + IVIg + methothrexate	NR: stable 6y after onset
[12] ^a^	steroids + cyclophosphamide + IVIg	NR: stable 4,5y after onset
[12]	none	NA
[12]	none	NA
[12]	none	NA
[12]	none	NA
[12]	none	NA
[12]	none	NA
[11] ^a^	plasmapheresis	NR
	steroids + azathioprine	R: increased muscle strength, myalgia disappeared, serum paraprotein level decreased; weakness relapsed after discontinuation; stable under long-term medication
[49]	steroids + azathioprine + mycophenolate mofetil	NR: stable for 2y
	rituximab + cyclophosphamide	R: increased muscle strength, followed by stabilization
[51] ^a^	auto-PBSCT + melphalan	R for 4 m, followed by further loss of muscle strength
	bortezomib + dexamethasone	R: muscle strength improved
	auto-PBSCT + melphalan	R: for 1y, followed by further loss of muscle strength
	bortezomib + cyclophosphamide + dexamethasone	R: clinical improvement, paraprotein in blood disappeared
[10] ^a^	plasmapheresis	PR: increased bulbar and neck strength, other muscles NR
	steroids + cyclophosphamide	PR: increased muscle strength + vital capacity, paraprotein level in blood decreased, patient still disabled
[38]	steroids + azathioprine	NR
[8]	steroids	NR
[52] ^a^	riluzole	NR
	IVIg	NR
[26] ^a^	steroids + mycophenolate mofetil	PR: increased muscle strength in legs, neck extensor weakness progressed
	IVIg, steroids + mycophenolate mofetil	R: slow modest increase of muscle strength for 6 months
[57]	posterior cervical orthodesis	R: improved head drop, minimal rotatory restriction
[13] ^a^	steroids + cyclophosphamide + IVIg + rituximab	NR: died 21 m after onset
[6]	NIV	condition stabilized, orthopnea disappeared, weakness remained stable
[31]	peridural infiltration	R: improvement of lumbar cruralgia, stable for 3y
[5]	steroids	NR
[58] ^a^	IVIg + steroids	NR
	auto-PBSCT + melphalan	R: increased muscle strength over 1y, further decrease in muscle strength after 2y
[25] ^a^	IVIg	R: increased muscle strength over 2y with almost complete resolution, stable after 3y therapy
[25] ^a^	IVIg + steroids + mycophenolate mofetil	R: increased muscle strength after 1y, but significant weakness remained
[27] ^a^	lenalidomide + dexamethasone	R: significantly increased muscle strength over 3 m, continuing for 4y
[48] ^a^	auto-PBSCT + melphalan	R: increased muscle strength over 1y, monoclonal protein level in blood undetectable
[3]	steroids	NR
[21]	steroids + azathioprine	NR
[50]	IVIg + steroids	R: increased muscle strength
[55] ^a^	steroids	NR
	auto-PBSCT + melphalan	R: increased muscle strength over 15 m
[1]	steroids	NR
[1] ^a^	steroids	NR
[4]	steroids	NR
[59] ^a^	steroids + azathioprine	PR: myalgia disappeared, but weakness progressed
[60]	steroids	R: increased muscle strength, relapse after discontinuation of therapy
[61]	steroids + azathioprine	NR
[29]	steroids + IVIg	NR: skin rash disappeared, weakness progressed
[62] ^a^	steroids + IVIg	NR
[63]	NIV	R: respiratory weakness resolved

*NIV* noninvasive ventilation, *NA* not available, *R* responsive, *PR* partially responsive, *NR* not responsive, *IVIg* intravenous immunoglobulins, *CK* creatine kinase, *auto-PBSCT* autologous peripheral blood stem cell therapy, *y* years, *m* months

^a^ Patients with monoclonal gammopathy

The most common treatment in SLONM patients was immunosuppressive therapy (62%), mainly consisting of steroids, with improvement of muscle strength in one third of the patients (Tables 1 and 2). Intravenous immunoglobulins (IVIg) were administered in 19%; in one third of these cases improvement in muscle strength was achieved. Plasmapheresis was performed in 3 patients, which led to a partial improvement in one of them. The most successful treatment in SLONM patients was high-dose melphalan followed by autologous peripheral blood stem cell transplantation (auto-PBSCT), resulting in an improvement in 6/7 treated patients (86%). This treatment was performed only in SLONM patients with MGUS. Noninvasive ventilation (NIV) was successful in 4 patients with respiratory insufficiency. Invasive ventilation was performed in one case [6]. All therapeutic regimens and outcomes in all patients for whom this information was available (*n* = 58) are listed in Table 2.

### Comparison between SLONM patients with and without MGUS (Table 1)

SLONM and MGUS affected significantly more males (*p* = 0.012). Age at onset did not differ significantly between the two groups (*p* = 0.34). SLONM patients with MGUS showed a significantly more rapid disease progression (*p* = 0.0010). Bulbar weakness resulting in dysphagia, dysarthria, and dyspnea was moderately, but not significantly more frequent in patients with MGUS (*p* = 0.20–0.36; Table 1). Muscle weakness in upper (*p* = 0.0064) and lower (*p* = 0.030) limbs was significantly more common in patients with MGUS. Although facial (*p* = 0.13), neck extensor (*p* = 0.38), trunk weakness (*p* = 0.63), and respiratory insufficiency (*p* = 0.35) were more commonly present in patients with MGUS, the differences were not statistically significant. Treatment response to immunosuppressive drugs (*p* = 0.69) or IVIg (*p* = 1.00) did not differ significantly between patients with and without MGUS. Plasmapheresis or melphalan followed by auto-PBSCT was only applied in patients with MGUS.

Muscle biopsy of 2 SLONM patients with MGUS revealed intranuclear rods, whereas this feature was not observed in patients without paraproteinemia. Vacuolar changes in muscle fibers were significantly more frequent in SLONM with gammopathy than without (*p* = 0.041). Myofibrillar disintegration (*p* = 0.21), inflammatory infiltrates (*p* = 0.64), fiber necrosis (*p* = 0.62), and core-like areas (*p* = 0.27) were noted more frequently in SLONM patients with MGUS. However, these differences did not reach significance (Table 1).

### Comparison between patients with SLONM and HIV-NM (Table 1)

There were significantly more males affected by HIV-NM compared to the SLONM cohort (*p* = 0.01). Age at onset of HIV-NM cases was significantly lower in comparison to SLONM patients (*p* = 0.01). No significant differences were noted in disease progression (*p* = 0.37) or in any of the clinical features (*p* > 0.05) except for the occurrence of myalgia in patients with HIV-NM (*p* = 0.043). Facial or respiratory weakness has not yet been reported in patients with HIV-NM. Immunosuppressive therapy (steroids) was administered more frequently in HIV-NM (*p* = 0.09), with a significantly more favorable outcome (*p* = 0.00071). The two muscle biopsy features that significantly distinguished HIV-NM from SLONM were the appearance of inflammatory infiltrates (*p* = 0.034) and fiber necrosis (*p* = 0.014). Interestingly, MGUS was detected in half of the HIV-NM patients, similar to the SLONM cohort.

According to the Classification of the United States Center for Disease Control and Prevention [32], the HIV-status in patients with HIV-NM was heterogeneous (infection stage 1 in five patients; stage 2 in two patients; stage 3 in one patient). The infection stage of the other eight patients remained unknown due to the absence of information in the literature regarding CD4-positive cell count and stage 3-defining opportunistic diseases.

### Genetic analyses using a targeted NGS gene panel (Additional file 1: Table S1)

The variants identified by targeted NGS, after applying a panel of 283 genes, in 6 SLONM patients without MGUS (Table 1) are summarized in Additional file 1: Table S1. We did not identify variants in any of the 11 known genes causing congenital nemaline myopathy (*TPM3, NEB, ACTA1, TPM2, TNNT1, KBTBD13, CFN2, KLHL40, KLHL41, LMOD3, MYPN*). In two patients (P2, P11), a distinct variant of unknown significance was identified in the ryanodine receptor 1 gene (*RYR1*). Both *RYR1* variants have not been reported before.

There were no common variants among the six SLONM patients in any of the genes tested. The variants identified were heterozygous and for the most part, of unknown significance (Additional file 1: Table S1). In P2 a known mutation was identified in *SCN11A* that has previously been associated with small-fiber neuropathy [33]. Our patient did not, however, show any symptoms or signs compatible with small-fiber neuropathy. In P10 a heterozygous variant in SH3TC2 was identified; however, no additional mutation could be found. Homozygous mutations in the *SH3TC2* gene cause autosomal recessive Charcot-Marie-Tooth neuropathy [34], heterozygous mutations can cause subtle autosomal dominant distal neuropathy [35]. In the current example, the patient did not show any signs of neuropathy.

## Discussion

The clinical presentation of progressive weakness and atrophy of proximal upper or lower extremity and axial muscles, accompanied by dyspnea and/or dysphagia, represented the predominant clinical phenotype of SLONM. MGUS was a major feature, present in half of the patients, which was not only associated with more rapid disease progression, but also with a favorable response to treatment, especially with melphalan/auto-PBSCT.

Neck extensor weakness, sometimes resulting in dropped head, occurred in more than half of the SLONM patients during their disease course. In six cases, dropped head was an initial symptom; accompanied by limb weakness in four, by dysphagia in one patient, and as an isolated finding in another case. Respiratory affection resulting in dyspnea was present in four patients at disease onset. Interestingly, 3 of these 4 patients with dyspnea as a presenting symptom did not suffer symptoms from insufficient breathing prior to presentation. This lack of respiratory symptoms is considered to be due to the slowness of respiratory decline in SLONM.

Most patients presented with myopathic or mixed EMG patterns. However, one published case [30] and one of our patients (P 3) showed a neurogenic EMG pattern. Neurogenic EMG patterns can appear in severe chronic myopathies (in so-called ‘end-stage’ muscle) [36, 37]. This could explain at least one of the two cases with a neurogenic EMG [30], as this patient died as the result of her disease one year after presentation. In contrast, the other patient (P 3) did not present a particularly severe chronic myopathy. However, this patient suffered from chronic lower back pains radiating in the legs, which may explain the neurogenic changes on EMG in this patient. An additional peripheral neuropathy was excluded by nerve conduction studies in both patients.

In fact, SLONM should be considered in the differential diagnosis of sporadic inclusion body myositis (sIBM), idiopathic inflammatory myopathies (IIM) and amyotrophic lateral sclerosis (ALS). ALS, similar to SLONM, can present similarly with dropped head, bulbar weakness, dyspnea and fasciculations. There is even more overlap between IIM and SLONM; however, the former usually presents with high serum CK levels, while the latter shows only slight elevations. Inflammatory infiltrates are usually a prominent histopathological feature of IIM, but can also appear in SLONM. However, among the IIMs, polymyositis and sporadic inclusion body myositis (sIBM) are characterized by direct infiltration of muscle fibers by cytotoxic T-lymphocytes immunoreactive for CD8, which was absent in our SLONM cases. Perifascicular muscle fiber atrophy and loss of endomysial capillaries, typically found in dermatomyositis, were also absent in our cases. Sporadic inclusion body myositis may share many clinical similarities with SLONM, including age at onset in late adulthood, weakness of the proximal lower limbs and dysphagia. However, predominantly distal involvement is uncommon in SLONM, in contrast to sIBM. Histopathologically, both sIBM and SLONM present a myopathic pattern, but we could not identify any inclusion bodies (tubulofilamentous inclusions), and only rarely rimmed vacuoles in the SLONM cohort [38, 39]. Furthermore, in patients with sIBM, nemaline rods do not appear within muscle fibers.

Rarely, SLONM has been reported in combination with thyroid dysfunction. In our study, TSH has been increased in one and decreased in another patient. Since both hyper- and hypothyroidism, as such, may cause myopathy [40], a direct impact of the change in thyroid hormones on muscle might contribute to the disease.

Several clinical clues, including age at onset, distinguish SLONM from congenital nemaline myopathies. Mean age at onset in the SLONM cohort was 52 years, whereas patients with hereditary nemaline myopathy usually develop symptoms during infancy or early childhood. Furthermore, perinatal hypotonia, delayed motor milestones, scoliosis and a high-arched palate are features of congenital nemaline myopathies and were therefore exclusion criteria for our study. Histopathologically, fiber type 1 predominance, which is typically seen in the congenital forms, was not present in the SLONM cohort. Both congenital and late-onset nemaline myopathies are characterized by the presence of rods in the muscle fibers [1, 41]. Our genetic analysis did not disclose any mutations in genes known to cause congenital nemaline myopathy. We did not include the very recently reported myopalladin gene (*MYPN*) in our gene panel, which in case of mutations was shown to cause nemaline myopathy as well [42]. Thus, mutations in *MYPN* were not searched for in our SLONM cohort. Furthermore, we did not perform an analysis of copy number variations. In intranuclear rod myopathy, which is a congenital nemaline myopathy with more severe disease course, nemaline rods are frequently found inside myonuclei [43]. Until recently, the only known causative gene was *ACTA1* [44]. However, very recently, mutations in the myopalladin gene (*MYPN*) were also found to cause intranuclear rods [44], in a relatively mild, childhood- to adult-onset nemaline myopathy with slowly progressive muscle weakness. The publication did not include any patients with SLONM that met our inclusion criteria, as three patients experienced first symptoms in their first decade and the fourth patient showed a positive family history for myopathy with consanguinity and neonatal muscular weakness. Interestingly, the muscle biopsy of two SLONM patients with MGUS and of two SLONM patients lacking immunoelectropheresis revealed intranuclear rods, whereas this feature was not seen in any of our SLONM patients without paraproteinemia. In all four cases with SLONM and intranuclear rods, nemaline bodies were also present in the sarcoplasm [4, 12, 21]. Genetic analysis of *ACTA1* was not performed in any of the four cases. The clinical phenotype of patients with SLONM with intranuclear rods was not significantly different compared to other patients with SLONM. In SLONM, nemaline rods are the most striking feature, but other unspecific changes such as fiber atrophy, internalization of nuclei, myofibrillar disintegration were also frequent. Mitochondrial changes, including ragged-red fibers, COX-negative fibers and/or paracrystalline intramitochondrial inclusions detectable by EM, occurred frequently (64%). These findings might be considered unspecific and age-related in part; however, a more direct effect of Z-disc remodeling on skeletal muscle fiber mitochondria cannot be excluded.

HIV-associated nemaline myopathy showed the same constellation of features seen in SLONM, including proximal limb weakness, myopathic EMG and slightly elevated serum CK levels. However, HIV-NM appears to be more confined to certain muscle groups, since neither facial nor respiratory involvement has been reported to date. Apart from one patient with dysphagia, bulbar weakness has not been described either. Interestingly, the majority of patients (73%) showed clinical signs of myopathy before the HIV infection was detected. Therefore, it is important to screen (especially younger) patients with SLONM for HIV infection. Of note, one case has even been described with negative serology at presentation with HIV seropositivity developing only a year after myopathy onset [45]. Histopathologically, there were marginal differences: rods were smaller with <1 μm radius [15, 46] and they did not appear inside the nucleus in HIV-NM. Moreover, we observed significantly more frequent myonecrosis and inflammatory infiltrates in HIV-NM. The most compelling difference, however, is the good clinical response of treated HIV-NM cases to immunosuppressive therapy. Combined with muscle involvement during the early stage of HIV infection, this finding might suggest either a direct impact of the viral particles on the formation of rods or an immunological trigger, rather than a degenerative mechanism due to T-cell suppression/immunodeficiency. An HIV-caused genome disturbance has also been discussed as a basis for rod formation [46].

In SLONM with MGUS, not merely the aggregation of paraproteins but rather their effects on autogenous antigens, especially sarcomeric proteins, are thought to cause the formation of rods [47, 48]. In fact, one case of SLONM with MGUS was described with sarcolemmal deposits of the paraprotein [10], and another suggested immunoreactivity of the sarcolemma to IgG with the paraprotein light chain [11], lending support to this theory. However, a similar immunohistochemical pattern was reported in another publication of a patient without MGUS [28], suggesting a similar pathogenic pathway in SLONM without MGUS. SLONM patients with accompanying autoimmune diseases such as systemic lupus erythematosus (SLE) [49] or Sjögren’s syndrome [50] often improved during immunosuppressive medication, whereas the majority of SLONM cases showed a disappointing response rate. This observation might hint at a different nonimmunogenic cause. Another hypothesis suggested that rod formation is part of a regenerative process without the traditional features of muscle repair [29]; however, our findings fail to support this assertion. The chronology of the appearance of rods in relation to disease course varied: Nine cases did not show any rods at symptom onset on light microscopy, but only in a subsequent biopsy (P 4) [8, 25, 26, 29, 49, 51–53]. Six cases revealed rods only after severe clinical deterioration (P4) [8, 25, 29, 51, 52]. Interestingly, this clinical correlation has also been observed the other way around: clinical improvement after auto-PBSCT was accompanied by the disappearance of rods [54, 55] as well as paraprotein reduction in SLONM patients with MGUS [56]. Therefore, multiple biopsies during the disease course may be necessary to confirm the diagnosis of SLONM.

Overall, the pathomechanisms of SLONM remain unsolved.

## Conclusions

To conclude, SLONM can be difficult to diagnose in routine clinical practice, not only because of its rarity, but also due to several clinical mimics. However, if an adult patient presents with progressive limb, bulbar and/or respiratory weakness, we recommend respiratory function testing as well as a muscle biopsy that includes a Gomori’s trichrome stain in order to identify nemaline rods. If rods are not visible by light microscopy, additional electron microscopic studies are indicated. In addition, all SLONM patients should be screened for a concomitant MGUS or HIV infection, since both have distinct therapeutic and prognostic implications.

## Additional file

Additional file 1: Table S1. Genetic variants identified in patients with SLONM using NGS and a panel of 283 genes. (DOC 72 kb)
