# Supplementary material for: Sporadic late-onset nemaline myopathy: clinico-pathological characteristics and review of 76 cases
Source: Orphanet J Rare Dis. 2017 May 11;12:86. doi: 10.1186/s13023-017-0640-2 (PMC5425967; doi:10.1186/s13023-017-0640-2)
Supplement: Additional file 1: Table S1. — Genetic variants identified in patients with SLONM using NGS and a panel of 283 genes. (DOC 72 kb) [file 13023_2017_640_MOESM1_ESM.doc]

| Additional file1: **Table S1: Genetic variants identified in patients with SLONM using NGS and a panel of 283 genes.** | | | | |
| --- | --- | --- | --- | --- |
| **Patient ID** | **Gene** | **Variant (all heterozygous)** | **Diseases known to be associated with the gene** | **Interpretation** |
| P 2 | *SCN11A* | c.3473T>C; p.Leu1158Pro | Familial episodic pain syndrome (OMIM #615552); hereditary sensory and autonomous neuropathy Type VII (OMIM #615548) | known variant (pathogenic) |
| P 2 | *RYR1* | c.10441-6T>C; p. ? | AD multicore myopathy with external ophthalmoplegia (OMIM #255320); central core myopathy (OMIM #117000); malignant hyperthermia (OMIM #145600); core-rod myopathy | unknown significance |
| P 2 | *COL6A1* | c.1182+3G>A; p. ? | Bethlem myopathy (OMIM #158810); AD or AR congenital Ullrich muscular dystrophy (OMIM #254090) | unknown significance |
| P 2 | *COL12A1* | c.4000+5G>A; p. ? | AD or AR myopathy (PMID 24334769 and 24334604) | unknown significance |
| P 2 | *TTN* | c.87949G>A; p.Ala29317Thr | AD tibial muscle dystrophy (OMIM #600334); Edström myopathy (OMIM #603689); cardiomyopathy (OMIM #613765); AR limb-girdle muscular dystrophy (OMIM #608807; AR myopathy (OMIM #611705) | unknown significance |
| P 2 | *BIN1* | c.1741C>T; p.Arg581Cys | AR centronuclear myopathy (OMIM #255200) | unknown significance |
| P 2 | *MSTN* | c.539T>C; p.Met180Thr | AR muscle hypertrophy (OMIM #614160) | unknown significance |
| P 2 | *PLEC* | c.1344+4A>T; p. ? | AR limb-girdle muscular dystrophy (OMIM #613723); muscle dystrophy with epidermolysis bullosa simplex (OMIM #22670); epidermolysis bullosa simplex with pyloric atresia (OMIM #612138); AR congenital myasthenic syndrome (PMID 21263134) | unknown significance |
| P 2 | *PNPLA6* | c.1484C>T; p.Pro495Leu | Boucher-Neuhäuser syndrome (OMIM #21547); Oliver-McFarlane syndrome (OMIM #275400); Laurence-Moon syndrome (OMIM #245800); cerebellar ataxia (PMID 25133958) | unknown significance |
| P 2 | *PRX* | c.1574T>C; p.Val525Ala | AR CMT neuropathy (OMIM #614895); Déjerine-Sottas syndrome (OMIM #145900) | probably benign |
| P 6 | *MYH14* | c.4318G>A; p.Val1440Met | Non-syndromal sensorineural hearing loss (OMIM #600652); AD peripheral neuropathy and myopathy (OMIM #614369) | unknown significance |
| P 6 | *PLEC* | c.172C>G; p.Arg58Gly | AR limb-girdle muscular dystrophy (OMIM #613723); muscle dystrophy with epidermolysis bullosa simplex (OMIM #22670); epidermolysis bullosa simplex with pyloric atresia (OMIM #612138); AR congenital myasthenic syndrome (PMID 21263134) | unknown significance |
| P 7 | *POLG2* | c.366G>T; p.Arg122Ser | AD progressive external ophthalmoplegia with mitochondrial DNA-deletions (OMIM #610131) | unknown significance |
| P 7 | *TIA1* | c.1070A>G; p.Asn357Ser | AR distal Welander myopathy (OMIM#604454) | unknown significance |
| P 7 | *TTN* | c.95984T>C; p.Val31995Ala | AD tibial muscle dystrophy (OMIM #600334); Edström myopathy (OMIM #603689); cardiomyopathy (OMIM #613765); AR limb-girdle muscular dystrophy (OMIM #608807; AR myopathy (OMIM #611705) | unknown significance |
| P 7 | *TTN* | c.92197G>A; p.Glu30733Lys |  | unknown significance |
| P 7 | *TTN* | c.13492C>T; p.Leu4498Phe |  | unknown significance |
| P 7 | *TTN* | c.12304C>A; p.Pro4102Thr |  | unknown significance |
| P 7 | *PYGM* | c.2009C>T; p.Ala670Val | AR McArlde myopathy (OMIM #232600) | unknown significance |
| P 7 | *ACADVL* | c.1844G>A; p.Arg615Gln | AR Acyl-Coenzyme-A-Dehydrogenase deficiency (OMIM #232600); cardiomyopathy(OMIM #201475) | unknown significance |
| P 10 | *MYH14* | c.3395C>A; p.Ala1132Asp | Non-syndromal sensorineural hearing loss (OMIM #600652); AD peripheral neuropathy and myopathy (OMIM #614369) | unknown significance |
| P 10 | *VMA21* | c.86C>T; p.Thr29Met | X-chromosomal recessive myopathy with excessive autophagia (ORPHA25980, PMID 256836990, PMID 25809233) | unknown significance |
| P 10 | *SH3TC2* | c.3686A>T; p.Asp1229Val | AR CMT neuropathy (OMIM #601596) | known variant  (only 1 variant in AR disease) |
| P 10 | *TTN* | c.36532+5T>C; p ? | AD tibial muscle dystrophy (OMIM #600334); Edström myopathy (OMIM #603689); cardiomyopathy (OMIM #613765); AR limb-girdle muscular dystrophy (OMIM #608807; AR myopathy (OMIM #611705) | unknown significance |
| P 11 | *RYR1* | c.9895G>A; p.Gly3299Ser | AD multicore myopathy with external ophthalmoplegia (OMIM #255320); central core myopathy (OMIM #117000); malignant hyperthermia (OMIM #145600); core-rod myopathy | unknown significance |
| P 11 | *DNA2* | c.689C>T; p.Ser230Leu | AD progressive external ophthalmoplegia Type 6; axial and proximal scelettal muscle weakness; myalgia; muscle cramps; AR Seckel syndrome Type 8 | unknown significance |
| P 11 | *MFN2* | c.2113G>A; p.Val705Ile | AR CMT neuropathy Type 2A; AD hereditary motor and sensory neuropathy | known variant  (polymorphism) |
| P 11 | *TTN* | c.88469G>A; p.Arg29490Gln | AD tibial muscle dystrophy (OMIM #600334); Edström myopathy (OMIM #603689); cardiomyopathy (OMIM #613765); AR limb-girdle muscular dystrophy (OMIM #608807; AR myopathy (OMIM #611705) | unknown significance |
| P 11 | *ACADM* | c.797A>G; p.Asp266Gly | AR middle-chain-Acyl-CoA-Dehydrogenase deficiency | unknown significance |
| P 11 | *MEGF10* | c.1841-5T>C; p. ? | early onset myopathy with areflexia | unknown significance |
| P 12 | *MYH2* | c.3181C>G; p.Leu1061Val | AD hereditary inclusion body myopathy Type 3 (OMIM #605637) Familial myopathy (PMID 15741996) | known variant (polymorphism) |
| P 12 | *TTN* | c.78272A>C; p.Lys26091Thr | AD tibial muscle dystrophy (OMIM #600334); Edström myopathy (OMIM #603689); cardiomyopathy (OMIM #613765); AR limb-girdle muscular dystrophy (OMIM #608807; AR myopathy (OMIM #611705) | unknown significance |
| P 12 | *TK2* | c.94C>T; p.Arg32Trp | AR myopathy with mitochondrial DNA-depletion syndrome (OMIM #609560) | unknown significance |
| *SCN11A*, sodium channel, voltage-gated, type XI, alpha subunit; OMIM, Online Mendelian Inheritance in Man; *RYR1*, ryanodine receptor 1; AD, autosomal dominant; *COL6A1*, collagen, type VI, alpha-1; AR, autosomal recessive; *COL12A1*, collagen, type XII, alpha-1; *TTN*, titin; *BIN1*, bridging integrator 1; *MSTN*, myostatin; *PLEC*, plectin; *PNPLA6*, patatin-like phospholipase domain-containing protein 6; *PRX*, periaxin; *MYH14*, myosin, heavy chain 14, nonmuscle; *POLG2*, polymerase DNA, gamma-2; *TIA1*, cytotoxic granule-associated RNA-binding protein; *PYGM*, glycogen phosphorylase, muscle; *ACADVL*, acyl-CoA dehydrogenase, very long-chain; *SH3TC2*, SH3 domain and tetratricopeptide repeat domain 2; *DNA2*, DNA replication helicase 2; *MFN2*, mitofusin 2;  *ACADM*, acyl-CoA dehydrogenase, medium-chain; *MEGF10*, multiple epidermal growth factor-like domains 10; *MYH2*, myosin, heavy chain 2, skeletal muscle, adult; *TK2*, thymidine kinase, mitochondrial. | | | | |
